# Accelerated Bone Healing via Electrical Stimulation

**DOI:** 10.1002/advs.202404190

**Published:** 2024-08-08

**Authors:** Jianfeng Sun, Wenqing Xie, Yuxiang Wu, Zhou Li, Yusheng Li

**Affiliations:** ^1^ Department of Orthopedics Xiangya Hospital Central South University Changsha Hunan 410008 China; ^2^ School of Kinesiology Jianghan University Wuhan Hubei 430056 China; ^3^ Beijing Institute of Nanoenergy and Nanosystems Chinese Academy of Sciences Beijing 101400 China; ^4^ National Clinical Research Center for Geriatric Disorders Xiangya Hospital Central South University Changsha Hunan 410008 China

**Keywords:** accelerated healing, bone defect, electrical stimulation, fracture, nanogenerators

## Abstract

Piezoelectric effect produces an electrical signal when stress is applied to the bone. When the integrity of the bone is destroyed, the biopotential within the defect site is reduced and several physiological responses are initiated to facilitate healing. During the healing of the bone defect, the bioelectric potential returns to normal levels. Treatment of fractures that exceed innate regenerative capacity or exhibit delayed healing requires surgical intervention for bone reconstruction. For bone defects that cannot heal on their own, exogenous electric fields are used to assist in treatment. This paper reviews the effects of exogenous electrical stimulation on bone healing, including osteogenesis, angiogenesis, reduction in inflammation and effects on the peripheral nervous system. This paper also reviews novel electrical stimulation methods, such as small power supplies and nanogenerators, that have emerged in recent years. Finally, the challenges and future trends of using electrical stimulation therapy for accelerating bone healing are discussed.

## Graphical abstract

Electrical stimulation significantly impacts bone healing by enhancing osteoblast proliferation, differentiation, and vascularization through calmodulin/calcineurin/NFAT signaling. It also boosts macrophage function and cell migration, presenting a comprehensive approach to accelerating bone repair. The novel self‐powered system using nanogenerators demonstrates significant potential for repairing bone defects, offering a promising therapeutic approach.

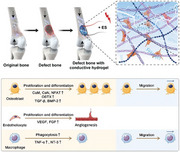

## 1 Introduction

Bone is a compact kind of connective tissue that consists of cellular components, extracellular matrix (ECM), and glial fibers. The skeletal structure can be classified into two distinct types of bone tissue, namely dense and cancellous bones. The primary characteristic that sets bone apart from other tissues is its notable abundance of calcium salts that are accumulated inside its ECM, hence enhancing its rigidity.^[^ ^1^ ^]^

Bones can be injured by various mechanisms, including trauma, infections, tumors, and impaired blood supply. Injured bone is replaced by new bone during the physiological process of bone healing, which returns the damaged bone to its pre‐injury mechanical and biological characteristics.^[^ ^2^ ^]^ This process involves complex interactions and the effects of mechanical forces in the biological environment near the injury site. After fracture, a hematoma is immediately formed, and an inflammatory reaction occurs. Platelets and macrophages migrate fracture sites and release inflammatory cytokines.^[^ ^3^ ^]^ Microscopic bridging of the fracture site takes place once the fracture ends are firmly consolidated and is facilitated by the intraosseous tissue, Haversian system, and periosteum.^[^ ^4^ ^]^ Intramembranous ossification takes place at the fracture ends without cartilaginous callus. Endochondral ossification occurs when the fracture ends are not in direct contact with each other or when the fracture site is relatively unstable. After the inflammatory phase, the new bone gradually replaces the cartilage callus.^[^ ^2^ ^]^ Many factors influence fracture healing. Internal factors encompass the nature and extent of trauma, local soft tissue injury, blood supply, differentiation potential of osteoprogenitor cells, and the cellular microenvironment. External factors include fracture fixation stability, spacing of the fracture ends, inflammation, and external stimuli. Even social habits, such as smoking and alcohol consumption, contribute to impaired fracture healing.^[^ ^5^ ^]^

The global prevalence of bone fractures is on the rise, mostly attributed to the health complications associated with the aging population and the gradual increase in average life expectancy. The United Nations research data estimates the number of individuals over the age of 65 to increase from 524 million in 2010 to ≈1.5 billion in 2050.^[^ ^6^ ^]^ As people age, the likelihood of fractures increases, especially fractures of the leg, wrist, and hip bones.^[^ ^7^ ^]^ Incidence of fractures is associated with the upper limb region, especially the distal radius and metacarpal regions, which account for 29.2% of the total number of fractures. Ankle and metatarsal regions and femur exhibit low extremity fractures.^[^ ^8^ ^]^ With the aging population, the incidence of fractures escalates, resulting in higher hospitalization and mortality rates among the elderly. This trend consequently drives up healthcare costs related to fracture treatment.^[^ ^9^ ^]^

Fracture healing is a critical healthcare issue. Impaired fracture healing, especially non‐healing, imposes medical costs on patients and healthcare systems for additional treatments, which are added to the indirect economic costs of incapacitation.^[^ ^10^ ^]^ The impact and incidence of fractures in 204 countries over 29 years are summarized in the Global Burden of Disease Study of 2019.^[^ ^11^ ^]^ The age‐standardized prevalence of fractures in 2019 was 2296.2 cases per 100 000 population. The global prevalence of long‐term fracture symptoms and the number of years lived with a disability increased by 70.1% and 65.3%, respectively. In the United States alone, traumatic fractures cost the economy $265.4 billion annually in medical costs and work time.^[^ ^12^ ^]^ Surgical intervention is necessary for the reconstruction of fractures that surpass the inherent regenerative capacity of the body or demonstrate delayed healing. In clinical practice, autografts are the current gold standard for treating these fractures and defects.^[^ ^13^ ^]^ In addition to surgical treatments, many efforts have been made to enhance and accelerate the fracture healing process. Adjunctive therapies involve electrical stimulation (ES) and low‐intensity pulsed ultrasound, low‐level laser therapy, mesenchymal stem cell therapy, and other modalities.^[^ ^14^ ^]^ ES and its association with bone formation were first reported by Fukada and Yasuda.^[^ ^15^ ^]^ They reported the piezoelectric properties of bone, that is, the generation of endogenous electric fields when bone is subjected to mechanical stress. Subsequently, researchers have made efforts to use exogenous electric fields to treat various bone injuries in animals and humans.^[^ ^16^ ^]^

## 2 Structure of the Bone Tissue

Bone tissue consists of three main cell types: osteoblasts, osteoclasts, and osteocytes. Osteoblasts originate from mesenchymal stem cells (MSCs) situated within the bone marrow.^[^ ^17^ ^]^ Their main function is to produce and deposit osteoid, which constitutes the unmineralized organic component of the ECM of bone.^[^ ^18^ ^]^ During the initial phases of human development, osteoblasts exhibit heightened levels of activity and produce bone matrix proteins, such as collagen type 1α1, osteocalcin (OCN), and alkaline phosphatase (ALP).^[^ ^19^ ^]^ Runt‐related transcription factor 2 (Runx2) regulates primary typing and differentiation. Osteoblasts mature into osteocytes in the mineralized bone matrix.^[^ ^20^ ^]^ Osteocytes comprise the primary constituents of bone tissue. Osteoclasts, derived from bone marrow precursors, are essential in bone resorption. This process is initiated by the stimulation of the macrophage colony‐stimulating factor and the receptor activator of nuclear factor kappa‐B ligand (RANKL).^[^ ^21^ ^]^ Bone tissue is abundant in ECM, which consists of 35% organic matrix and 65% inorganic mineral matrix in terms of volume.^[^ ^22^ ^]^ The organic matrix is mainly composed of type I collagen fibers (90%) and the rest is composed of various proteoglycans (biglycan, lumican, and osteoadherin) and glycoproteins (OCN, osteopontin, and osteonectin).^[^ ^23^ ^]^ The inorganic mineralization of the bone matrix consists mainly of nano‐hydroxyapatite (Ca10 (PO4)6 (OH)2), along with small amounts of magnesium, fluorine, and manganese salts, which provide most of the stiffness.^[^ ^24^ ^]^

## 3 Endogenous Electric Field of the Bone

The endogenous physiological field of cells constitutes the basis of all cellular physiological processes.^[^ ^25^ ^]^ The arrangement, migration, proliferation, and differentiation of osteoblasts are influenced by any changes in the exogenous electric and magnetic fields.^[^ ^26^ ^]^ In the last century, researchers have identified the piezoelectric effect in bone; when stress is exerted on the bone, it produces electrical signals. Electrical signals in the bones arise from collagen.^[^ ^27^ ^]^ When the electrical signal induces stimulation in bone, it subsequently triggers the activation of membrane proteins located on the cell surface, as well as Ca^2+^ voltage‐gated channels on the cell membrane surface. This process leads to changes in the intracellular and extracellular Ca^2+^ concentrations. Direct current (DC) stimulation has been found to induce the secretion of prostaglandin E2 (PGE2), morphological substances, and growth factors, thereby exerting an influence on cellular processes.^[^ ^28^ ^]^ Therefore, ES exerts positive effects on the bone tissue.^[^ ^29^ ^]^

In bone tissue, an electric field surrounds the endogenous cells, including osteoblasts, osteocytes, and osteoclasts, which arises from the transmembrane potential. This is due to the difference in intracellular and extracellular ion concentrations (K^+^, Na^+^, and Ca^2+^), estimated to be in the range of 40–500 mV mm^−1^.^[^ ^30^ ^]^ Voltage‐gated Ca^2+^ ion channels play a role in regulating osteogenesis, osteoblast and osteoclast functions, and bone regeneration. Voltage‐gated Ca^2+^ channel mediates the influx of Ca^2+^ ions into osteoblasts during ES, which further enhances osteogenic differentiation by upregulating the transforming growth factor (TGF)‐β1 levels mediated by calmodulin.^[^ ^31^ ^]^ Enhanced osteogenesis is primarily mediated by voltage‐gated Ca^2+^ channels, although ES also activates voltage‐gated Na^+^, K^+^, and Cl^−^ channels.^[^ ^32^ ^]^ When osteoclasts are subjected to ES, influx of Ca^2+^ ions mediated by the Ca^2+^ channel of the voltage gate leads to changes in the cytoskeleton, hindering podosome expression in osteoclasts, which further inhibits bone resorption in these cells.^[^ ^33^ ^]^

## 4 Exogenous ES Accelerating Bone Healing

The osteogenic function of ES in fracture healing was originally described by Fukada et al.^[^ ^15^ ^]^ ES has proven to be effective in promoting bone formation during bone repair, including unassociated fractures, osteoporosis, and osteonecrosis.^[^ ^6^, ^34^ ^]^ In the clinical environment, three different methods are used for ES: DC, pulsed electromagnetic field (PEMF), and capacitive coupling (CC). DC stimulation is performed via surgically implanted ES power supplies and electrodes with a current dose of between 10 and 100 mA.^[^ ^35^ ^]^ Both the CC and pulsed PEMFs are applied externally. In CC, an alternating voltage is applied to the skin electrodes placed on both sides of the fracture to generate an electric field of 0.1–20 G.^[^ ^36^ ^]^ Alternating current in the current‐carrying coil produces PEMF on the skin of the fracture site, resulting in a peak‐to‐peak range of 3–10 V at the fracture site.^[^ ^37^ ^]^ Here, we summarize various electrical stimuli and their effects (**Table** **1**) and discuss their effects on bone healing.

**Table 1:** Summary of bone healing via electrical stimulation using traditional power supply.

Device	Type of current	Signal characteristics	Animal or cell model	Effect of bone healing	Ref.
Orthopulse	PEMF	Pulse amplitude: 50 mV Pulse width: 5 µs Burst width: 5 ms Burst refractory period: 62 ms Time: 8 h/d Frequency: 15 Hz	Human (postoperative delayed union of long‐bone fracture)	–	[44]
EBI bone healing device	PEMF	Burst width: 4.5 ms Peak magnetic field: 1.2 mT Frequency: 15 Hz	Human umbilical vein endothelial cells (HUVECs)	Stimulate the endothelial release of fibroblast growth factor 2 (FGF‐2) and induce paracrine and autocrine changes in the surrounding tissues	[50b]
EBI bone healing device	PEMF	Burst width: 4.5 ms Peak magnetic field: 1.8 mT Frequency: 15 Hz	Fetal rat calvarial (FRC) cells	Increase osteoblast proliferation	[50c]
Interdigitated electrodes (IDEs)	DC	Voltage: 500 mV mm^−1^ Time: 3 h	MC3T3 cells	Promote proliferation and differentiation of MC3T3 cells	[71]
IonOptix	PEMF	Voltage: 1 V Pulse duration: 3.6 ms Electric field: 90 V m^−1^ Current: 12 mA Frequency: 7.9 Hz	Human osteoblast‐like MG‐63 cells	Affect osteoblast adhesion and calcium ion signaling	[73c]
BIOSET	DC	Current: 10 µA Time: 5 min (twice per week)	Rats (defect of the calvary bone)	Modulate the Wnt pathways and accelerates osteogenesis with improved tissue maturation	[79b]
Biostim	PEMF	Impulse length: 1.3 ms^−1^ Peak magnetic field: 2.8 mT Time: 24 h Frequency: 75 Hz	Horses (metacarpals)	Affect the expression of inflammatory cytokines (tumor necrosis factor [TNF]‐α and interleukin [IL]−6)	[61]
Trio 300	DC	Current: 1 mA Time: 15 min (thrice every six days) Frequency: 200 Hz	Rats (defect of the parietal bone)	Raise the parathyroid hormone‐intact (PTH‐i) level in the blood to activate osteoclasts during the early stage of bone remodeling	[94]
Fracture healing patch (FHP)	PEMF	Pulse intensity: 0.05–0.5 mT Pulse frequency: 20 KHz Cycle frequency: 10 Hz Time: 24 h	Human (acute distal radius fractures)	Promote osteoblast differentiation and maturation	[106a]
OrthoPak	CC	Voltage: 3–6 V Current: 5–10 mA Time: 15–20 h Frequency: 60 kHz	Human (acute tibial stress fracture)	Increase activated calmodulin levels	[106b]
Biolectron Inc	CC	Voltage: 3–6.3 V Current: 5–10 mA Time: 24 h Frequency: 60 kHz	Human (stress fractures)	Stimulate and augment the bone tissue potentials directly involved in new bone production and repair	[106c]

### 4.1 DC Stimulation

**Figure 1:**
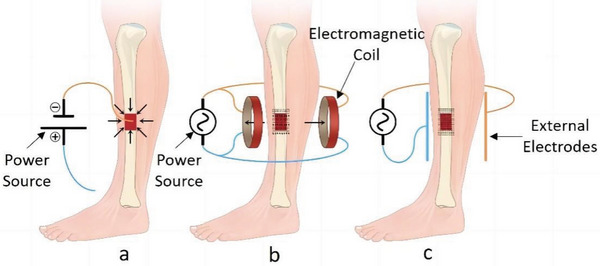
Electrical bone growth stimulation, classified by mechanism of operation and electrical energy delivery. a) Direct current electrical stimulation. b) Inductive coupling. c) Capacitive coupling.

Direct current ES (DCES) is an invasive technique that involves the implantation of an electrode into the bone (**Figure** **1**a). The positioning of the cathode at the location of bone injury induces an electrochemical decrease of molecular oxygen, resulting in the formation of an alkaline and hypoxic microenvironment.^[^ ^38^ ^]^ This state is conducive to the differentiation of osteoblasts and activates osteoclasts to generate vascular endothelial growth factors, thus inducing angiogenesis.^[^ ^39^ ^]^ It also increases the ratio of osteoblasts to osteoclasts.^[^ ^40^ ^]^ Simultaneously, DC increases proteoglycan and collagen. Exogenous DC stimulates cell surface receptors coupled with phospholipase C (PLC) and increases intracellular Ca^2+^ concentrations in human osteoblasts. This process facilitates the activation of PLC, resulting in the synthesis of inositol triphosphate, which subsequently interacts with intracellular receptors, triggering the release of Ca^2+^.^[^ ^41^ ^]^ Microcurrent enhances transcriptional activation of genes related to Hedgehog, TGF‐β1, and mitogen‐activated protein kinase(MAPK) signaling pathways.^[^ ^42^ ^]^ TGF‐β1 plays a crucial role in osteoblasts, and its mRNA expression is regulated by the calcium/calmodulin pathway.^[^ ^31c^ ^]^ Therefore, ES promotes the multi‐step process of osteoblast mineralization.

DCES is not the best method for treating acute fractures because of the need for surgery to place leads and the risk of infection or equipment failure. Faradic reactions at the electrodes can partially inhibit osteogenesis. Safe current densities and charges for bone healing are limited.^[^ ^43^ ^]^ Consequently, DCES is only occasionally employed for acute fractures with high risks of delayed or non‐union.

### 4.2 Inductive Coupling (IC)

PEMF therapy is an attractive modality for fracture healing due to its non‐invasive nature, eliminating any risk of infection or requirement of additional surgery^[^ ^44^ ^]^ (Figure 1b). PEMF therapy activates intracellular voltage‐gated calcium channels to increase cytoplasmic calcium. PEMF enhances bone marrow stem cells (BMSCs) differentiation in the early stages of osteogenesis by increasing the expression of L‐type voltage‐gated calcium channels and cytoplasmic calcium concentration.^[^ ^45^ ^]^ And it upregulates insulin‐like growth factor 2, bone morphogenetic proteins [BMPs]−2 and 4, and TGF‐β to induce osteoblast proliferation, differentiation, and ECM deposition.^[^ ^14^, ^38^ ^]^ PEMF promotes the proliferation and differentiation of mesenchymal stem cells, and stimulates bone morphogenetic proteins and the formation of bone scabs through the production of calcium calmodulin, phospholipase A2, the synthesis of PGE2, and other ingredients that aid healing.

PEMF improved trabecular microstructure and increased osteogenic differentiation of osteoblasts by activating the Wnt/β‐catenin pathway, leading to osteogenesis.^[^ ^46^ ^]^ PEMF also upregulates the classical Wnt ligands Wnt1, 3a, 10b, LRP5 and LRP6, which are associated with increased bone mass and strength. Recently, Wu et al. showed that both Wnt classical and non‐classical signaling pathways are involved in osteogenic differentiation.^[^ ^47^ ^]^ Notch signaling is required for skeletal progenitor cells in the process of fracture repair.^[^ ^48^ ^]^ Bagheri et al. showed that PEMF regulates the same Notch genes implicated in osteogenesis and cooperates with the osteogenic microenvironment via the Notch pathway.^[^ ^49^ ^]^

Angiogenesis is an important process in the formation of new bone. PEMF can increase angiogenesis and perfusion in many bone‐related models.^[^ ^50^ ^]^ The mechanism by which PEMF stimulates angiogenesis seems to depend fibroblast growth factor 2 (FGF2). Goto et al. reported that compared to control mice, the expression levels of angiopoietin 2 and fibroblast growth factor 2 were increased in the femurs of mice treated with a PEMF.^[^ ^51^ ^]^

### 4.3 CC

CC entails the application of electrodes onto the skin surface to establish an electric field amidst the electrodes (Figure 1c). Proliferation of osteocytes induces an elevation in the intracellular calcium concentration because it transfers calcium into the cell. Signal transduction results in the transfer of calcium through the channel, which subsequently leads to increased prostaglandin and calmodulin levels. In addition, CC increases BMP and noggin levels.^[^ ^52^ ^]^ CC activates voltage‐gated calcium channels to increase cytosolic calcium levels, thereby initiating the calmodulin‐mediated osteogenic pathway.^[^ ^52^ ^]^ CC therapy is also related to the upregulation of BMP2, 4, and TGF‐β1 in osteoblasts.^[^ ^31^, ^53^ ^]^ Under alternating current, the electrochemical reaction continues without the risk of faradic salts accumulation. Thus, the charge density is much higher than that of DC. The limited ability of the electric field to penetrate soft tissue restricts the applicability of CC to superficial bones, such as the distal radius.^[^ ^54^ ^]^

Although all electric fields can generate potentially effective electric fields in tissues, capacitively coupled electric field stimulation (CCEFS) has marked advantages over DC or PEMF in stimulating bones. DC stimulation is an invasive method of treatment. PEMFs generate electromagnetic fields from coils and use a heavy power supply that requires daily charging. In contrast, the capacitively coupled electric field device is small and lightweight (4 ounces); it uses a battery and a gel electrode. CCEFS can stimulate osteocytes to produce more DNA than PEMF. The difference in effect may be that PEMF depends on activating limited intracellular calcium storage, whereas CCEFS utilizes unlimited calcium in the outer space of the cells.^[^ ^52^ ^]^

The current view is that the mechanical load on the bone is transferred to osteocytes through fluid flow in the bone tissue caused by strain. Membrane shear stress is a tangential force produced by fluid flow that can induce actin reorganization into stress fibers and increase the expression of c‐fos and cyclooxygenase‐2 following inositol‐triphosphate‐mediated intracellular calcium release,^[^ ^55^ ^]^ similar to the increase in activated calmodulin observed by CCEFS.^[^ ^52^ ^]^ The detection of flow potentials originating from the surface of a loaded bone serves as empirical evidence of fluid flow occurring within the bone cortex. Electric field stimulation of the bone may exert its effects through fluid flow electroosmosis (an electrokinetic force, as opposed to a flow potential). Hence, the flow of electrolytic bone fluid could be the result of an electric field applied to the bone.

## 5 Effects of ES on Bone Healing

Here, we present the effects of ES on bone healing in terms of reduction of inflammation, osteogenesis, angiogenesis and the effects on osteoclasts and peripheral nervous system.

### 5.1 Reduction of Inflammation

When bone is destroyed, a hematoma forms within the injured bone, resulting from bleeding in the fractured bone and from vessels beneath the periosteum.^[^ ^56^ ^]^ Biochemical messengers that induce an inflammatory response are released. They regulate the protein synthesis, migration, and proliferation of cells that are crucial to osteoblast activation, bone remodeling and angiogenesis.^[^ ^57^ ^]^ In particular, interleukin 6 (IL‐6) participates in the differentiation of osteoblasts and osteoclast progenitors and is expressed throughout the healing process of fractures.^[^ ^57a^ ^]^ Tumor necrosis factor (TNF)‐α facilitates the activation of osteoclasts and the subsequent resorption of bone,^[^ ^58^ ^]^ whereas TGF‐ β is recognized as the potent fibrinogen cytokine,^[^ ^59^ ^]^ playing a crucial role in tissue repair processes.^[^ ^60^ ^]^ PEMFs can affect the expression of TNF‐α and IL‐6 during the initial phases of bone healing.^[^ ^61^ ^]^

The bidirectional interactions between immune cells and bone cells play a crucial role in bone remodeling and bone healing.^[^ ^56^ ^]^ The early inflammatory phase of bone healing has been identified as a viable target for immunomodulatory treatments to promote bone repair in bone immunology.^[^ ^62^ ^]^ The immune system assumes a critical function as the first responder to host injury. Macrophages are quickly recruited to the injured site to initiate the inflammatory response.^[^ ^63^ ^]^ Earlier studies have shown that DCES does not change the phenotype of macrophages,^[^ ^64^ ^]^ while recent studies have shown that ES causes anodic migration of macrophages and cathodic migration of monocytes, which contributes to the localization and initiation of these cells and enhances bone healing. ES also significantly improved macrophage phagocytosis and selectively regulated cytokine production. Electric field exposure moderately increased the production of TNF‐a. Neurotrophin‐3 is a definite healing medium related to M2 macrophages that increases significantly.^[^ ^65^ ^]^ ES stimulates osteogenesis by upregulating the transcription of osteogenic genes (Spp2 and Bmp2) in macrophages.^[^ ^66^ ^]^ Low‐voltage ES activates Vsig4 (M1 inhibition) and Pla2g5 gene (M2 development) to alter the response of macrophages by changing the ratio of M1 to M2 macrophages.^[^ ^67^ ^]^ Taken together, ES exerts a notable impact on these macrophage subsets. The above‐mentioned study was to use ES to modulate macrophages and other immune cells to promote regeneration.

### 5.2 Enhancement of Osteogenesis

**Figure 2:**
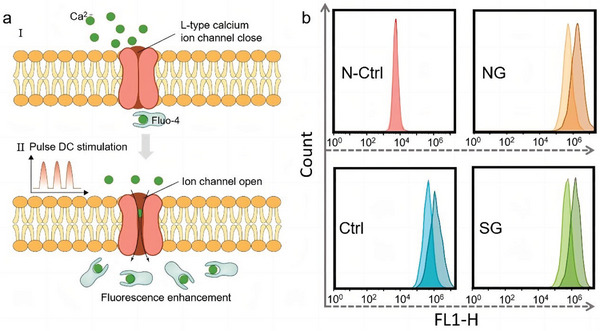
Intracellular calcium ion level after different electrical stimulation periods. a) Schematic of intracellular calcium concentration promoted by electrical stimulation; calcium ion concentration was characterized via fluorescence staining. b) Intracellular Ca^2+^ was measured using a flow cytometer in the same group after electrical stimulation for 1 and 3 days. Unstained cells were used as N‐Ctrl.^[^ ^122^ ^]^ Copyright 2021, Elsevier.

Osteoblast differentiation and proliferation can be induced by ES. It has been discovered that the use of electrical currents ranging from 5 to 100 microamps to promote bone formation have beneficial effects. Osteoblasts secrete several bioactive substances to regulate and affect bone formation and reconstruction.^[^ ^68^ ^]^ Through ES of an electret‐based host‐coupling bio‐nanogenerator, calcium ion channels are activated, further activating the calmodulin (CaM)/calcineurin (CaN)/nuclear factor of activated T cells (NFAT) signaling pathway. CaM, a calcium ion binding protein, activates CaN expression and dephosphorylates p‐NFAT. NFAT is then introduced into the nucleus to initiate related signaling pathways and induce the expression of subsequent osteogenic proteins.^[^ ^69^ ^]^ Calcium has been discovered to be significant in bone cell response to ES. ES increases intracellular calcium ions concentration by releasing calcium from the intracellular calcium pool or by opening L‐type calcium ion channels, allowing extracellular calcium to flow into the cell^[^ ^70^ ^]^ (**Figure** **2**). Cytoplasmic calcium activates protein kinases or calmodulin to convert electrical signals into biological signals that promote gene expression and protein synthesis, affecting cell proliferation and differentiation.

RUNX2 and OSTX are the two primary transcription factors that are involved in the process of osteoblast differentiation. RUNX2 acts early and was first detected in the preosteoblasts. Its expression was upregulated in immature osteoblasts but not in mature osteoblasts.^[^ ^71^ ^]^ Increased OSTX expression indicates the onset of osteoblast maturation and differentiation. Electrically stimulated cultures showed higher levels of maturation and differentiation, exhibiting higher OSTX levels than other conditions. There were no significant changes in the expression levels of RUNX2, indicating no alterations during the early stages of maturation. These results may indicate that changes occur in the early stages of stimulation and regulate osteogenesis activated by ES through other molecules.^[^ ^66^ ^]^

ES promotes the differentiation of stem cells into osteoblasts, accompanied by an increase in the cell spreading area.^[^ ^72^ ^]^ ES also has a direct effect on cell adhesion and cell spreading.^[^ ^73^ ^]^ Cell spreading area affects cell differentiation within a certain range. The greater the area of cell spreading, the more effective the osteogenic induction.^[^ ^74^ ^]^ Cells with large spreading area have high cytoskeletal tension and activation of the nuclear Yes‐associated protein/transcription coactivator with PDZ‐binding motif (YAP/TAZ), which is important for the differentiation of MSCs.^[^ ^75^ ^]^ Cytokines also promote bone tissue healing, and bone morphogenetic proteins, such as BMP2, BMP7, and BMP9, can promote osteogenic or chondrogenic differentiation of MSCs.^[^ ^76^ ^]^ It has been reported that the transforming growth factor‐β (TGF‐β) family affects bone development and remodeling through different signaling pathways.^[^ ^77^ ^]^

VEGF and FGF are major mechanisms tightly coupled to angiogenesis and osteogenesis. In addition, more blood vessels were found in areas with higher VEGF and FGF1 expression, suggesting that fracture ES device(FED) can promote vascularization during bone healing. Enhanced secretion of TGF‐β and BMP2 can rapidly initiate bone remodeling, leading to high bone density and bone strength.^[^ ^78^ ^]^ In osteogenesis, surface charges can absorb beneficial proteins, form an ECM layer, promote cell deposition and tissue remodeling, and simultaneously trigger multiple molecular transduction mechanisms, such as calcium signaling, TGF‐β/BMP, MAPK/ERK, Wnt/β‐catenin pathway, etc. to induce osteogenesis.^[^ ^79^ ^]^

### 5.3 Enhancement of Angiogenesis

In some studies of skin wounds, ES stimulated the formation of new blood vessels into ischemic wounds from pre‐existing blood vessels in adjacent tissues.^[^ ^80^ ^]^ DCES promotes angiogenesis in vascular endothelial cells and regulates the synthesis of important growth factors and cytokines in angiogenesis via VEGF receptors.^[^ ^81^ ^]^ In a rat femoral large‐area defect model, using electrical stimulation for the bone defect treated with bone tissue engineering (BTE) significantly increased the formation of new blood vessels at the defect site.^[^ ^82^ ^]^ Research suggests that fracture healing may occur through endogenous electrical currents that target blood vessels at injury sites. In long‐term ununited bone fissures, the sequence of endochondrosis is interrupted during the fibrocartilage and vasculogenesis stages.^[^ ^83^ ^]^ Application of exogenous PEMFs provides long‐lasting electrical signals that enhances bone healing.

Tepper et al. ^[^ ^50b^ ^]^ showed that, after PEMF stimulation of vascular cells, the levels of basic fibroblast growth factor (bFGF‐2) and several other vascular growth factors (angiopoietin‐2, thrombopoietin, and epidermal growth factor) increased; however, VEGF‐A levels did not increase. PEMF enhances angiogenesis by stimulating vascular endothelium to release FGF‐2, which induces paracrine and autocrine secretion within the adjacent tissues. Similar to the low‐dose PEMF currently used clinically, it can significantly increase the proliferation and tubulization of endothelial cells, which are important processes in angiogenesis. PEMF stimulates endothelial cells to release proteins in a paracrine manner and upregulate angiogenesis. Therefore, it is likely that PEMF increases vascularization to promote complex fracture healing.^[^ ^50b^ ^]^

Activation of MAPK cascades is the main cellular signal transduction pathway that controls specific mRNA transcription in response to external stimuli such as ES.^[^ ^84^ ^]^ MAPK is a serine/threonine kinase protein that controls intracellular metabolic processes in reaction to extracellular stimuli.^[^ ^84^ ^]^ Protein kinases mediate many significant cell biological responses in these cascades, including proliferation, differentiation, and apoptosis, depending on the ES timing and the cell types.^[^ ^85^ ^]^ The activation of MAPK induced by ES was recorded in endothelial angiogenesis and HL‐60 differentiation.^[^ ^85^, ^86^ ^]^ Mechanically, the cell movement and wound healing responses induced by current gradients are dynamically regulated by phosphoinositide 3‐kinase and phosphate and tensin homolog signaling.^[^ ^87^ ^]^ The phosphorylation of extracellular signal‐regulated kinase, p38 MAPK, Src, and Akt at the Ser 473 site was accelerated and increased gradually in the cells subjected to electric shock.^[^ ^86^ ^]^ A low‐intensity current of 0.1 ms has been reported to transiently activate the p38‐p53 pathway, which may have an important impact in tumor eradication and downregulation of inflammatory cytokine responses. The effects of ES on stromal cell orientation and motility are associated with activation of the PI3K and ROCK signaling pathways.^[^ ^88^ ^]^

### 5.4 Effect on Osteoclasts

Interactions between osteoblasts and osteoclasts are important to regulate the formation of new bone.^[^ ^89^ ^]^ Osteoblasts and osteoclasts regulate each other's maturation at different phases of the bone remodeling process. In the early stages of bone remodeling, communication between osteoblasts and osteoclasts promotes pre‐osteoclast differentiation. Mature osteoclasts are accountable for absorbing compromised and aging bone tissue.^[^ ^90^ ^]^ Subsequently, osteoclasts stimulate the process of new bone growth by further activating osteoblasts.^[^ ^91^ ^]^ It is worth noting that the imbalance between osteoclast absorption and osteoblast regeneration during bone remodeling may lead to other bone diseases such as secondary osteoporosis.^[^ ^92^ ^]^ These findings indicate that osteoclast resorption and osteoblast regeneration are key factors in the process of bone healing.

Activating osteoclasts in the early stages of bone remodeling is beneficial for the removal and absorption of damaged bone tissue.^[^ ^93^ ^]^ Electroacupuncture requires regular repetition to produce a lasting effect. After four weeks of repeated electroacupuncture, the concentration of parathyroid hormone in the blood increased, which could activate osteoclasts and promote the autocrine function of osteoclasts at the injured site. Osteoclasts express p38 at 4–8 weeks, suggesting that osteoclasts perform bone resorption around bone defects. Therefore, electroacupuncture may control the increase in osteoclast number and early bone resorption by regulating the secretory mechanism of parathyroid hormone‐intact (PTH‐i)‐p38. Electroacupuncture increases PTH‐i levels in the blood to activate osteoclasts in the early phases of bone reconstruction. In addition, osteoclasts promote the maturation of osteoblasts in mid‐ and late‐stage to facilitate new bone regeneration.^[^ ^94^ ^]^

Acidic environment inhibits osteoblast proliferation, differentiation, and calcium absorption, while enhancing the activity of osteoclasts.^[^ ^95^ ^]^ Activation of osteoclasts and osteoblasts in relatively acidic and alkaline environments has been demonstrated by the measurement of osteoclast tartrate‐resistant acid phosphatase and osteoblast ALP levels. Therefore, the relatively alkaline environment of the cathode may provide appropriate conditions for osteoblast maturation.^[^ ^96^ ^]^ Calcium ions are important in promoting the differentiation of osteoclasts into active mature osteoclasts.^[^ ^97^ ^]^ ES directly activates voltage‐gated calcium channels on the cell membrane and increases intracellular calcium ion levels.^[^ ^70^ ^]^

### 5.5 Effects on the Peripheral Nervous System

Bone tissue is innervated by dense sensory neural networks, and the most common sensory nerve is the calcitonin gene‐related peptide (CGRP) positive nerve.^[^ ^98^ ^]^ CGRP‐positive nerves regenerate themselves in the process of fracture healing.^[^ ^99^ ^]^ CGRP is released after depolarization of neurons and promotes angiogenesis and osteogenesis.^[^ ^100^ ^]^ The damage of CGRP release from the injury site can delay fracture healing, resulting in delayed union or even non‐union,^[^ ^101^ ^]^ while CGRP supplementation can enhance regeneration at bone defects.^[^ ^100^, ^102^ ^]^ Therefore, CGRP plays a crucial role in fracture healing and holds potential as a therapeutic target for promoting fracture healing.

**Figure 3:**
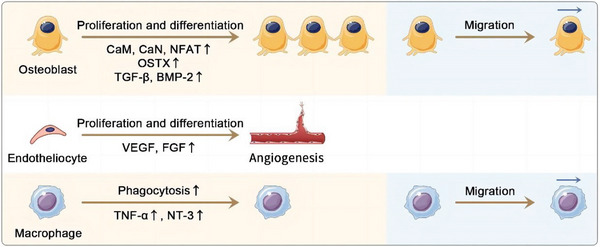
Effects of electrical stimulation on bone healing at cellular and tissue levels. Electrical stimulation could accelerate the proliferation and differentiation of osteoblasts by activating the calmodulin/calcineurin/nuclear factor of activated T cells signaling pathway and increasing the expression of OSTX, TGF‐β and BMP‐2. And electrical stimulation could promote the expression of VEGF and FGF to accelerate vascularization. Electric field exposure increased the production of TNF‐a and Neurotrophin‐3 to improve macrophage phagocytosis. And the osteoblast and macrophage migration could promote by electrical stimulation.

ES upregulates the CGRP synthesis in the dorsal root ganglia (DRGs) by activating the Ca2+/CaMKII/CREB signaling pathway, and rapidly releases CGRP through an action potential that triggers the vesicle bank of nerve terminals.^[^ ^103^ ^]^ ES in the DRGs upregulates CGRP biosynthesis, leading to its subsequent release in the femoral region. After being released from the fracture site, CGRP promotes the formation of H‐type blood vessels that couple angiogenesis and osteogenesis. Previous studies have reported that an increased abundance of H‐type vessels significantly promotes fracture healing and spinal fusion.^[^ ^104^ ^]^ In the process of endplate ossification in a model of painful intervertebral discs, the spatial correlation between H‐type vessels and CGRP‐positive nerves has been determined^[^ ^105^ ^]^(**Figure** **3**).

## 6 ES Therapy Devices and Methods for Bone Healing

The majority of clinical ES devices are conventional ES devices. Conventional ES devices, which mainly include DC, PEMF, and CC, have demonstrated favorable outcomes when used in bone healing therapy.^[^ ^106^ ^]^ However, ES devices are bulky for clinical treatments. Portable and patient‐compliant ES therapy devices for bone healing are still a great challenging. Portable and novel self‐containing ES devices based on nanogenerators (NGs) have been proposed.^[^ ^107^ ^]^ NG technologies include triboelectric NGs,^[^ ^108^ ^]^ piezoelectric NGs,^[^ ^109^ ^]^ thermoelectric NGs, and hybrid and coupled NGs that convert biomechanical energy into electrical energy. Examples are listed in **Table** **2**.

**Table 2:** Summary of bone healing via electrical stimulation using new‐type power supply.

Device	Type of current	Signal characteristics	Animal or cell model	Effect of bone healing	Ref.
Host‐coupling bio‐nanogenerator (HCBG)	DC	Voltage: 40 V Current: 0.98 µA Frequency: 1.2 Hz	Rats (femoral shaft)	Upregulate cytosolic calcium ion levels and activate the calcium‐sensing receptors by increasing the calcium ion influx	[69]
Pulsed triboelectric nanogenerator (P‐TENG)	Pulsed current	Current: 30 µA Frequency: 1.5 Hz	Bone marrow mesenchymal stromal cells (BMSCs)	Rejuvenate senescent BMSCs by enhancing MDM2‐dependent p53 degradation	[72c]
Bone fracture ES device (FED)‐TENG	Biphasic electric pulses	Voltage: 4.5 V	Rats (tibia fractures)	Activate relevant growth factors to regulate the bone microenvironment to promote bone formation and bone remodeling and accelerate bone regeneration and maturation	[78]
Bulk piezoelectric nanogenerators (BPENGs)‐PWH‐750	DC	Voltage: 8.24 V Current: 27.3 nA	Mouse preosteoblast (MC3T3‐E1) cells	Enhance osteogenic differentiation	[107b]
Hyaluronic acid (HA)‐triboelectric nanogenerators (HA‐TENGs)	DC	Voltage: 20 V Current: 0.4 µA	MC3T3 cells	Promote the proliferation of MC3T3‐E1 cells	[113]
Self‐powered electrical stimulator (TENG)	Pulsed direct current (DC, rectified)	Voltage: 100 V Current: 1.6 µA	MC3T3‐E1 cells	Promote the adhesion, proliferation, and differentiation of osteoblast progenitor cells and upregulates the calcium ion levels in them	[121]
sm‐PENG	Pulse‐DC	Current: 20 µA Frequency: 3 Hz Time: 2 h	Murine calvarial preosteoblasts (MC3T3‐E1, ATCC CRL‐2594)	Promote osteoblast differentiation	[122]

Wang et al. originally proposed NGs based on the self‐powered system of piezoelectric and triboelectric effects in 2006.^[^ ^110^ ^]^ NGs have proven to be effective applications of Maxwellian displacement currents in energy harvesters and sensors.^[^ ^111^ ^]^ NGs can be classified into piezoelectric NGs (PENGs) and triboelectric NGs (TENGs). A PENG consists of piezoelectric materials, flexible substrates, and electrodes.^[^ ^112^ ^]^ TENG was created by combining electrostatic induction and triboelectrification between various materials.^[^ ^113^ ^]^ NGs possess the unique characteristics of being self‐sustaining, easily transportable, adaptable, wearable, inexpensive, and possessing a high level of security. The utilization of self‐powered ES devices that depend on NGs is a feasible approach for wound treatment.^[^ ^112^ ^]^ As the output performance of NGs continues to improve,^[^ ^114^ ^]^ highly stable, low‐cost, lightweight, and easy‐to‐fabricate NGs show great potential in biomedical engineering for ES^[^ ^115^ ^]^ and biosensors^[^ ^116^ ^]^ including drug delivery, cancer treatment,^[^ ^117^ ^]^ nerve stimulation,^[^ ^118^ ^]^ muscle stimulation^[^ ^119^ ^]^ and health monitoring.^[^ ^120^ ^]^ It has also garnered considerable interest within the field of bone repair.^[^ ^121^ ^]^

**Figure 4:**
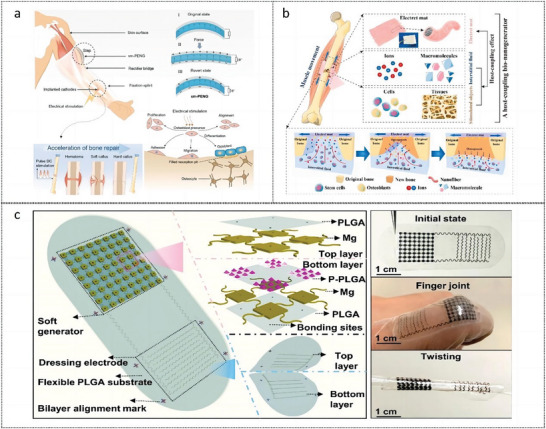
Nanogenerators for electrical stimulation of bone healing. a) Fixation splint with biomechanical‐energy‐driven shape memory piezoelectric nanogenerator to promote bone repair.^[^ ^122^ ^]^ Copyright 2021, Elsevier. b) An electret‐based host‐coupling bio‐nanogenerator implanted onto the bone injury in vivo.^[^ ^69^ ^]^ Copyright 2021, Elsevier. c) An implantable and ultraflexible bone fracture ES device based on triboelectric nanogenerators.^[^ ^78^ ^]^

Zhang et al. have devised a novel device for promoting osteogenic differentiation by ES. This device combines a shape‐memory compression‐based, arch‐shaped electrical NG (sm‐PENG) with a fracture fixation splint, enabling self‐powered electrical pulse DC stimulation for bone healing^[^ ^122^ ^]^ (**Figure** **4**a). sm‐PENG can increase cell proliferation and cellular ALP activity, promoting calcium deposition, mineralization, and osteogenic differentiation. The device has a broad spectrum of possible applications in the field of bone restoration. Yu et al. developed an electret‐based host‐coupled bionanogenerator (HCBG) for the ES of osteogenesis (Figure 4b). The implanted material is converted to electrical stimulation by harvesting biomechanical energy. Tissue fluids, cells, tissues, and organs at the host target site assume the role of the electrodes and circuits of the biogenerator. Upon implantation, the pads bind to the interstitial fluid and stimulate the host object to form HCBG. During the activity of muscle groups, HCBG scavenges biomechanical energy and activates osteogenesis by ES.^[^ ^69^ ^]^ Yao et al. proposed an implantable super‐flexible FED made entirely of biodegradable and bioabsorbable metal materials (Figure 4c). TENG components are designed with island bridge electrodes and pyramid‐shaped microstructural arrays for superior flexibility and considerable electrical output.^[^ ^78^ ^]^ FED can attach to irregular surfaces and generate steady electrical pulses in reaction to knee‐joint motion. Treatment results are comparable to those of clinical ES. Mechanistic studies have shown that the electric field generated by an FED can mimic the release of various growth factors and promote osteoblast proliferation, thereby promoting bone formation, reconstruction, and mineralization.

**Figure 5:**
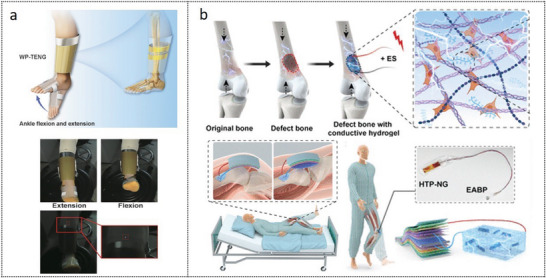
Wearable devices for bone defects repair. a) A wearable pulsed TENG enhanced the osteogenic differentiation ability of BMSCs and facilitated the repair and regeneration process of bone defects.^[^ ^123^ ^]^ Copyright 2022, John Wiley and Sons. b) Self‐powered electrical stimulation system based on hybrid tribo/piezoelectric NG and conductive hydrogel for bone defects repair.^[^ ^124^ ^]^

A wearable pulsed TENG was designed, which is capable of generating consistent pulsed electrical stimulation by harnessing human motion (**Figure** **5**a). ES elevated the intracellular Ca^2+^ concentrations, controlled the activity of transcription factors, and enhanced the expression of genes associated with the formation of bone tissue. Furthermore, TENG enhanced the osteogenic differentiation ability of BMSCs and facilitated the repair and regeneration process of bone defects.^[^ ^123^ ^]^ A self‐powered ES system was designed and fabricated using hybrid tribo/piezoelectric NG (HTP‐NG) and conductive hydrogel to repair bone defects (Figure 5b). The HTP‐NG is capable of efficiently capturing the energy from joint motion and simultaneously producing biphasic electrical pulse signals. The system has the capability to increase calcium ion influx and foster osteogenic differentiation, which in turn facilitates bone generation.^[^ ^124^ ^]^

In addition, there are several other methods to promote bone healing based on nanomaterials and NGs. Tang et al. developed a self‐powered low‐level laser curing system for osteogenesis using the integration of TENG and an infrared laser irradiation unit, which promotes osteoblast proliferation and differentiation, improves osteoclast mineralization, and accelerates bone healing.^[^ ^79a^ ^]^ Ultrasound has been shown to have a promising role in accelerating fracture healing.^[^ ^125^ ^]^ Piezoelectric nanofibers were utilized in combination with noninvasive ultrasound by researchers to generate a stable surface charge that promotes bone regeneration. Piezoelectric nanofiber tissue scaffolds can generate the desired charge under acoustic pressure to directly stimulate osteogenesis and repair defects.^[^ ^126^ ^]^

## 7 Effects of ES on Different Therapeutic Needs

In the field of oncology, ES exhibits distinctive therapeutic potential. DCES in the tumor area triggers electrolytic processes, generating electrochemically toxic products that impact the survival environment of tumor cells.^[^ ^127^ ^]^ Furthermore, the electroporation technique increases cell membrane permeability, enabling more accurate and effective delivery of therapeutic agents into tumor cells.^[^ ^128^ ^]^ ES disrupts intracellular homeostasis, adjusts the expression levels of the crucial tumor suppressor factor P53, and impedes the mitosis process, consequently inducing programmed cell death in tumor cells.^[^ ^129^ ^]^ Additionally, ES induces an immune response, activating targets of the immune response, inflammatory cells, and accumulating immunocyte factors, thereby enhancing the cytotoxic effects on tumor cells.^[^ ^130^ ^]^ ES has been demonstrated to reduce bacterial growth.^[^ ^131^ ^]^ ES can reduce bacterial infections and circumvent antibiotic resistance by altering bacterial cell membrane permeability through electroporation effects.^[^ ^132^ ^]^ The formation of high levels of hydrogen peroxide through high‐voltage electric field electrolysis is also an antibacterial mechanism of ES.^[^ ^133^ ^]^ In peri‐implantitis, ES impedes bacterial adhesion and proliferation, effectively preventing biofilm formation.^[^ ^134^ ^]^ In osteoporosis management, PEMF utilizes the RANKL/OPG and Wnt/β‐catenin pathways for bone mass restoration and inhibits osteoclastogenesis and differentiation to retard osteoporosis progression.^[^ ^135^ ^]^ Neuromuscular electrical stimulation enhances insulin sensitivity and reduces blood glucose levels in patients with type‐2‐diabetes mellitus.^[^ ^136^ ^]^

Within the same species, the parameters of ES required for different therapeutic needs, such as antitumor, antimicrobial, and angiogenesis, vary significantly. Additionally, even under the same therapeutic requirement, the parameters of ES differ due to the use of different devices or variations in the treatment site. (**Table** **3**) ES is associated with minimal side effects, usually manifesting as mild discomfort or pain due to muscle contractions near the electrodes. Other potential reactions, such as minor erythema and edema, may occasionally be noted.^[^ ^137^ ^]^

**Table 3:** Summary of different therapeutic needs via electrical stimulation.

Therapeutic need	Type	Signal characteristics	Species	Effect of bone healing	Ref.
Antitumor	Electric field	Voltage: 1 V Tissue conductivity: 0.5 S cm^−1^ Frequency: 100 kHz	Mice (B16F10)	Increase intracellular ROS production, and produce potent ICDs to activate the systemic immune response	[127b]
	Nano‐Pulse Stimulation	Peak voltage: 30 kV Current: 65–80 A Pulse width: 100 ns	Mice (C57BL/6)	Induce an immune response against the tumor driven by one or more neo‐antigens	[130a]
	Nanosecond pulsed electric fields	Peak voltage: 30 kV Pulse width: 100 ns Frequency: 1 Hz Duration: 200 pulses of 100 ns	Mice (C57BL/6)	Activate targets of immune respones, accumulation of inflammatory cells and immune cytokines	[130b]
	Nanosecond pulsed electric field	Peak voltage: 40 kV Frequency: 1 Hz Duration: 500 pulses of 100 ns	Canines	Alter electrical conductivity and permeability of the tumor cell membrane	[128c]
	Electrical pulse	Voltage: 1500 V Aspired current: 20 to 35 A Pulse length: 90 µs Duration: 80 pulses	Human (hepatocellular carcinoma)	Create nanopores in the cell membrane and lead to cell membrane disruption	[128b]
Antimicrobial	AC	Voltage: 0.5–4.5 V Current: 5–40 nA	Mice (S. aureus)	Accumulate electrical breakdown effect and produce H2O2	[133b]
	DC	Voltage: 1.75 V Duration: 24 h	Long‐Evans rats	Modify the local microenvironment to disrupt the adherent biofilm and reduce bacterial viability	[132b]
	DC	Voltage: 8–10 V Current density: 1 mA cm2	E. coli (SM2029)	Formate chlorine radicals and other consecutively formed RCS	[131b]
	AC	Voltage: 3.29 × 10^−2^ V Current: 6.5 mA	S. aureus	Disrupt bacterial cell membrane and block proliferation of bacterial cells	[134]
	Electric field	Voltage: 500 V Current: 60 µA	S. aureus and E. coli	Generate hydrogen peroxide and electroporation	[133c]
Angiogenesis	Electrical pulse	Voltage: 20–80 V Current: 0.004 mA Frequency: 60 Hz	Human	Increase VEGF‐A and PLGF expression	[81c]
	DC	Voltage: 1.2 V Current: 0.1–0.2 µA	Mice	Increased vessel density	[82]
	DC	Current: 10 mA Frequency: 1000 Hz	Sprague‐Dawley rats	Reduce oxidative stress, inflammation and apoptosis	[80c]
	PEMF	Magnetic field: 12 G Frequency: 15 Hz	Mice	Release protein in a paracrine fashion to induce changes in neighboring cells and up‐regulate angiogenesis	[50b]

## 8 Discussion and Outlook

Bone plays a crucial role in facilitating movement, providing structural support, and safeguarding vital organs, which are essential for the proper functioning of the human organism. Fracture healing takes a long time and is easily disturbed by many factors, resulting in delayed or even non‐healing, which significantly impacts the patient's quality of life. The efficacy of ES physiotherapy in facilitating fracture healing has been identified. This review outlines the role of ES in bone healing and summarizes the devices and methods available for ES. Most cells involved in bone healing are electrically responsive, making ES an important method to promote bone healing. Although different types of ES promote bone healing, several key issues need to be addressed to enhance their efficacy.

### 8.1 Accuracy and Personalization

Individual differences are observed among different ES bone‐healing treatment strategies, such as the treatment of different fracture sites and fresh/delayed fracture healing/non‐union. Moreover, the etiology and characteristics of different fractures exhibit variations. Therefore, individual characteristics and wound differences must be considered when using ES treatments. Future research should focus on improving the ES type, intensity, frequency, and duration.

### 8.2 Combination of Treatments

Autologous bone grafting is widely regarded as the preferred method for enhancing bone formation.^[^ ^138^ ^]^ In‐depth study of fracture‐healing mechanisms has resulted in various adjunctive therapies being used in clinical practice. Bone tissue engineering (BTE) mimics autologous bone grafting in many ways using scaffolds and osteoblasts to fill the defective bone and cell–cell and cell–scaffold interactions modulated by the addition of growth factors or ES.^[^ ^138^, ^139^ ^]^ Both traditional and adjuvant treatments significantly affect bone healing. Different treatments have different mechanisms for bone healing and combining multiple treatments may lead to better outcomes. However, the specific effects and mechanisms of these combined treatments require further investigation.

### 8.3 Self‐Powered Devices

NGs are self‐powered devices that are capable of converting mechanical energy into electrical energy. NGs are better at harvesting low‐frequency mechanical energy than electromagnetic induction and have great potential for harvesting distributed energy. Compared with electromagnetic induction, NGs exhibit superior performance in harvesting low‐frequency mechanical energy and have significant potential for harvesting distributed energy.^[^ ^140^ ^]^ NGs are widely used in wearable and implantable electronics as power sources and sensors.^[^ ^141^ ^]^ Biodegradable NGs can also be prepared via the rational use of materials. In addition to NGs, many other self‐powered devices, such as photovoltaics and pyroelectrics,^[^ ^142^ ^]^ are available that provide alternate solutions for ES therapy. Skillful and rational design of self‐powered ES devices and their application for bone healing should be further explored in the future.

## Conflict of Interest

The authors declare no conflict of interest.
